# Risk association of *RANKL* and *OPG* gene polymorphism with breast cancer to bone metastasis in Pashtun population of Khyber Pakhtunkhwa, Pakistan

**DOI:** 10.1371/journal.pone.0276813

**Published:** 2022-11-08

**Authors:** Faiza Hayat, Najeeb Ullah Khan, Aakif Ullah Khan, Iftikhar Ahmad, Ahmad M. Alamri, Bushra Iftikhar

**Affiliations:** 1 Institute of Biotechnology and Genetic Engineering (Health Division), The University of Agriculture, Peshawar, Khyber Pakhtunkhwa, Pakistan; 2 Institute of Radiotherapy and Nuclear Medicine (IRNUM), Peshawar, Khyber Pakhtunkhwa, Pakistan; 3 Research Center for Advanced Materials Science (RCAMS), King Khalid University, Abha, Saudi Arabia; 4 Khyber Medical College Peshawar, Peshawar, Pakistan; Universita degli Studi della Campania Luigi Vanvitelli, ITALY

## Abstract

**Introduction:**

The receptor activator NF-κB ligand (*RANKL*) and Osteoprotegrin (*OPG*) single nucleotide polymorphisms (SNPs) have been associated with the risk of breast cancer to bone metastasis. This study was designed to investigate the association of *RANKL* and *OPG* gene polymorphisms with breast to bone metastasis in Pashtun population of Khyber Pakhtunkhwa, Pakistan.

**Materials and methods:**

A total of 215 participants were enrolled containing 106 breast cancer patients, 58 breast to bone metastasis and 51 age and gender matched healthy controls. *RANKL* (rs9533156) and *OPG* (rs2073618, rs3102735) polymorphisms were genotyped in genomic DNA, using Tetra-ARMS PCR protocol. The results were analyzed among the three groups and *P*-value less then 0.05 were considered statistically significant.

**Results:**

Our results displayed significant association of *OPG* (rs3102735) risk allele and corresponding genotypes in breast cancer vs healthy controls, bone metastasis vs healthy controls and breast cancer vs breast to bone metastasis as a disease risk. However, there was no association observed for *OPG* (rs2073618) risk allele and corresponding genotypes with the diseases risk. Similarly, *RANKL* (rs9533156) risk allele and corresponding genotypes in breast cancer vs healthy controls, bone metastasis vs healthy controls and breast cancer vs breast to bone metastasis exhibited significant association except for the risk allele carrying genotypes in breast to bone metastasis.

**Conclusion:**

*OPG* (rs3102735) and *RANKL* (rs9533156) exhibited significant association with breast to bone metastasis while *OPG* (rs2073618) didn’t show significant association with breast to bone metastasis in Pashtun population of Pakistan. However, this study unlocks more questions to investigate the exact scenario of genetic predisposition of breast to bone metastasis.

## Introduction

Breast cancer is the most frequently diagnosed cancer in women worldwide [1] and one of the leading cancers in Asian countries, including Pakistan [2]. Breast cancer is regularly metastasizing to various organs such as bone (60–80%), lung (57–77%), liver (5–12%) and brain (10–30%) [3–6]. The process of metastasis is a multi-step sequential cascade starting primary tumor local invasion, progression towards lymphogenic spread and colonization at distant sites [7]. Genetic factors may predispose the probability of breast cancer metastasize to bone [8]. The discovery of the apoptosis regulator receptor activator NF-κB ligand (*RANKL*) and osteoclastogenesis inhibitor *OPG* signaling pathway exhibited major regulatory system for osteoclast formation and also *OPG/RANKL* alliance controlled the bone remodeling [9, 10], contributed significantly to the physiological bone turnover and also involved in osteoclast differentiation and activation [11].

*RANKL-OPG* pathway contributes to the primary tumorigenesis, bone metastasis and regulated by factors including progesterone and prolactin. Skeletal diseases caused by excessive osteoclastic activity including breast cancer to bone metastasis, rheumatoid arthritis and osteoporosis were triggered by imbalance between *RANKL* and *OPG* genes [12–15]. In addition, the *RANKL* and *OPG* also involved in proliferation of malignant and benign breast tumors [9]. Over expression of *OPG* by cancerous cells, increases tumor growth and cell proliferation [16]. *OPG* genetic variants are associated with bone mineral density, bone turnover and osteonecrosis [17–19].

*RANKL* polymorphism located in the proximal promoter interacts with transcription factors such as heat shock proteins, E2F1 (E2F transcription factor 1), vitamin D3 and core-binding factor a1 (Cbfa1), which affect the expression of *RANKL* gene [20, 21]. *RANKL* rs9533156 (T>C) causes DNA hypermethylation leading to gene silencing and transcriptional inactivation, that affects the binding of transcriptional factors [22, 23]. *OPG* rs2073618 influence secretions from the cells and drastically affect the secretory kinetics [10].

*RANKL/OPG* pathway suggests an important effect on the pathogenesis of breast cancer due to functional properties of genes. Genetic variation within *OPG* (rs3102735) promoter region and *RANKL* (rs9533156) near 5’ region could have an effect on the expression of genes and may have an influence on the development of tumors [10, 12]. Various studies conducted have elucidated the association of *RANKL* and *OPG* polymorphism with the breast cancer to bone metastasis risk in different ethnicities [24]. However, unfortunately, there is no such work done on Pakistani population practically the Pashtun population of Khyber Pakhtunkhwa, Pakistan. Here this study aimed to investigate the association of above-mentioned polymorphisms with breast cancer to bone metastasis in the patients of Pashtun population of Khyber Pakhtunkhwa, Pakistan.

## Materials and methods

### Study design, ethical approval and sampling

This case-control study was conducted at the Institute of Biotechnology and Genetic Engineering (IBGE), The University of Agriculture Peshawar (UAP), Pakistan. Patients were recruited from the Institute of Radiotherapy and Nuclear Medicine (IRNUM) Peshawar, Pakistan. The study protocol and informed consent (patients proforma) were approved (IBGE/UAP/2020/003) by the Ethical Committee of IBGE, UAP. Written informed consent was taken from all the participants while explaining the aim and objectives of the study. A total of 215 participants were enrolled, with age ranging from 25–80 years, containing (106 breast cancer patients only, 58 breast cancer to bone metastasis, and 51 healthy controls) from January 2021–December 2021. The demographic, clinical parameters and blood samples were collected from all the subjects through venipuncture procedure using sterile syringe. The blood was stored at– 20ºC in EDTA tubes at IBGE until further process.

### DNA isolation, SNP genotyping and gel electrophoresis

DNA was extracted from the whole blood using salting out (non-enzymatic) method and SNP genotyping was confirmed via T-ARMS PCR, previously adopted in our lab [25, 26]. Specific primers were designed using primer blast software for each SNP (Table 1). The PCR mixture (10μL) was prepared, consisting of 5μL master mix (Thermo Fisher Scientific, DreamTaq Master Mix (2X), 0.5μL of each forward and reverse primer, 3μL of ddH_2_O and 1μL of template DNA. The PCR condition were, initial denaturation at 95°C for 5min. followed by 35 cycles, denaturation at 95°C for 30 sec. annealing for 30 sec. at 61°C, 63°C and 60°C for *OPG* rs2073618, rs3102735 and *RANKL* rs9533156 respectively, extension 72°C for 30 sec. and final extension at 72°C for 7min. The amplified PCR products were run and confirmed using 1.5% gel electrophoresis with 1KB DNA ladder (Thermo Fisher Scientific).

**Table 1:** Specific primer sequences used for genotyping.

Genes (SNP)	Primers	Sequence 5’-3’
*OPG* (rs2073618), C/G	Forward outer	AGCCTAACCCCAAGCCTC
	Reverse outer	CCCTGGGGGATCCTTTCC
	Forward inner	AGCGCGCAGCACAGCAAC
	Reverse inner	TCCGGGGACCACAA
*OPG* (rs3102735), T/C	Forward outer	CGTACCCGGCTGCCTGAC
	Reverse outer	CCGGGTACGGCGGAAACT
	Forward inner	GTTCGCTGTCTCCCCCATCTGAACAAC
	Reverse inner	GTCTAACTTCTAGACCAGGGAATTA
*RANKL* (rs9533156), TC	Forward outer	TCAGCAACTTCCTTCTGAAGAAGT
	Reverse outer	AGTCTCGGTTTCCTTAGGATTTAGA
	Forward inner	CCCTTTACCCTTTTCTCTGCACC
	Reverse inner	CTGACTTTATAAAGATGAAAACTCCA

### Statistical analysis

SPSS software (version 2.0) was used for statistical analysis. Demographic data was analyzed via descriptive statistical method and *p*<0.05 was considered significant. Chi-square test was performed for categorical data, while t-test and one-way ANOVA was applied for analysis of quantitative data. The genotypic and allelic frequency was calculated by using chi-square and Fisher exact test. Online software named Medcalc was used for obtaining odd ratios and 95% CI.

## Results

### Demographic and clinicopathological parameters association with diseases risk

A total of 215 participants were recruited in this study, consisting of (breast cancer patients, BC = 106, breast to bone metastasis, BM = 58, and healthy controls, HC = 51). The demographics and clinical characteristics association was checked among the study groups (Table 2). The results showed no significant association among the ages (*p* = 0.811), tumor size (*p* = 0.268), stage (*p* = 0.067), HER2 status (*p* = 0.284), PR status (*p* = 1.00), ER status (*p* = 0.940) and Luminal subtypes (*p* = 0.639). However, significant association was observed in menstrual status (*p* = 0.0001), nulliparous (*p* = 0.0001), family history (*p* = 0.0001), localization (*p* = 0.0001), Ki67 (*p* = 0.0001), histology (*p* = 0.0001), nodal status (*p* = 0.0001) and IHC subtypes (*p* = 0.0001).

**Table 2:** Demographics, clinical and pathological parameters and their association with the disease risk.

Characteristics	BC n (%)	BM n (%)	HC	*p*-value
Total	106	58	51	
Age Group (years)	45.811±10.477	55.55±11.948	41.6274±6.627	0.811
Menstrual Status				
Premenopausal	43 (40.56)	19 (32.76)	36 (70.59)	0.0001
Postmenopausal	63 (59.44)	39 (67.24)	15 (29.41)	
Nulliparous				
No	97 (91.51)	49 (84.48)	-	0.0001
Yes	9 (8.49)	9 (15.52)		
Family History				
Negative	92 (86.79)	46 (79.31)	-	0.0001
Positive	14 (13.21)	12 (20.69)		
Localization				
Left	51 (48.11)	37 (63.79)	-	0.0001
Right	55 (51.89)	21 (36.21)		
Ki67				
<14%	37 (34.91)	2 (3.45)	-	0.0001
>14%	69 (65.09)	56 (96.55)		
Tumor Size				
T1 (<2cm)	14 (13.21)	3 (5.17)	-	0.268
T2 (2.1–5 cm)	59 (55.66)	36 (62.07)		
T3 (>5 cm)	33 (31.13)	19 (32.76)		
Stage				
2	2 (1.87)	0 (0)	-	0.067
3	33 (31.13)	12 (20.69)		
4	71 (66.98)	46 (79.31)		
Histology				
ILC	6 (5.66)	15 (25.86)	-	0.0001
IDC	100 (94.34)	43 (74.14)		
Her 2 Status				
Negative	64 (60.38)	30 (51.72)	-	0.284
Positive	42 (39.62)	28 (48.28)		
PR Status				
Negative	53 (50)	29 (50)	-	1.00
Positive	53 (50)	29 (50)		
ER Status				
Negative	50 (40.17)	27 (46.55)	-	0.940
Positive	56 (52.83)	31 (53.45)		
Nodal Status				
Negative	38 (35.85)	12 (20.69)	-	0.0001
Positive	68 (64.15)	46 (79.31)		
IHC Subtypes				
Luminal A	35 (33.02)	15 (25.86)	-	0.0001
Luminal B	23 (21.69)	15 (25.86)		
Her2 enriched	20 (18.87)	16 (27.59)		
TNBC	28 (26.42)	12 (20.69)		
Luminal subtypes				
Luminal A + B	58 (54.72)	30 (51.72)	-	0.639
Non-Luminal				
Her2 enriched + TNBC	48 (45.28)	28 (48.27)	-	

### *OPG* (rs3102735) and *RANKL* (rs9533156) exhibited significant association with breast cancer risk while *OPG* (rs2073618) didn’t show association compared with healthy controls

**Fig 1:**
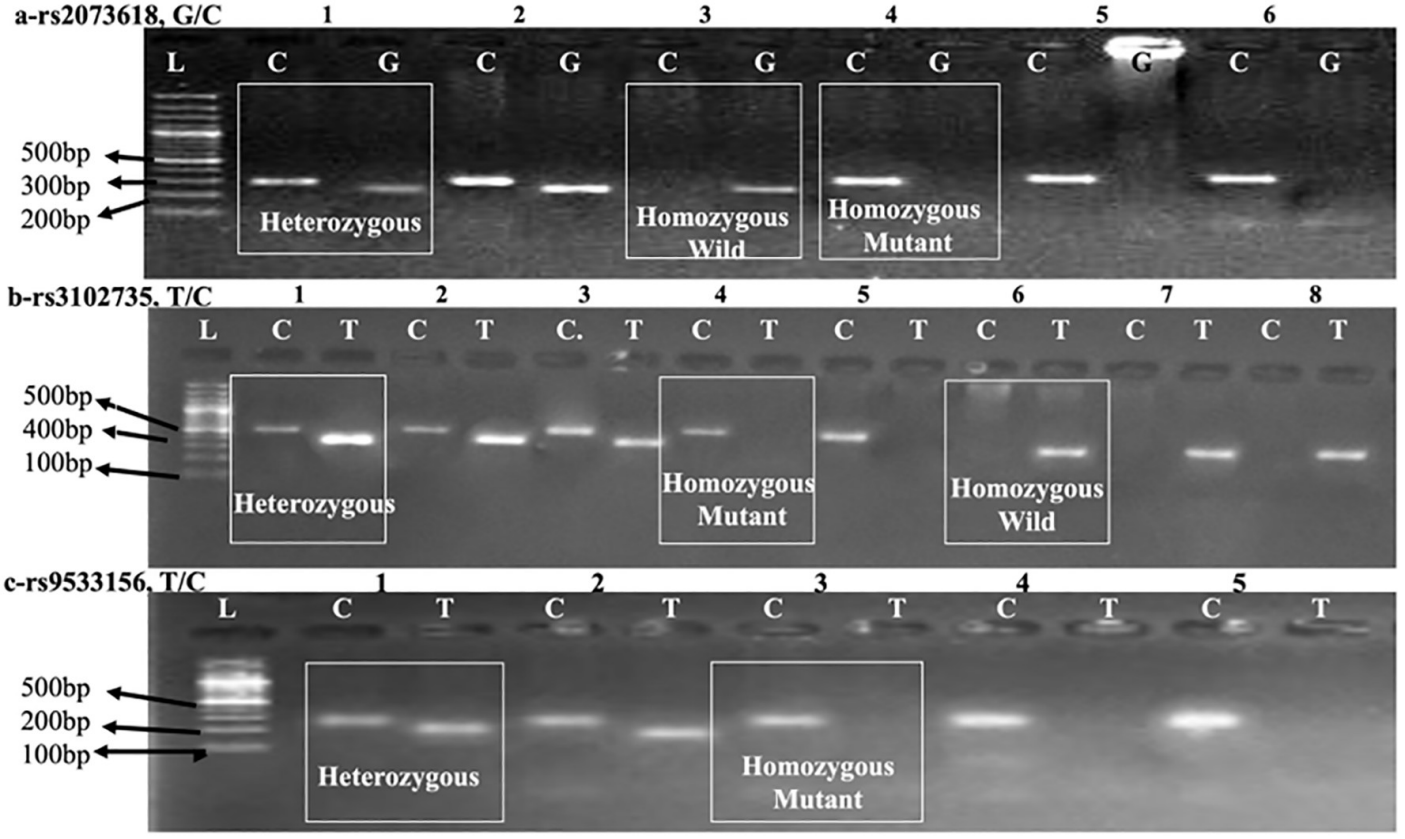
Electrophoretogram showing T-ARMS PCR based amplification of random samples (a) *OPG* (rs2073618), (b) *OPG* (rs3102735), (c) *RANKL* (rs9533156), L, 1KB DNA ladder (Thermo Fishier Scientific).

All the samples were analyzed for SNPs genotyping using T-ARMS-PCR, run and confirmed on 2% agarose gel with 1KB DNA ladder (Fig 1). The results indicated that *OPG* (rs2073618) risk allele C (OR: 1.2293 CI: 0.7651–1.9751, *p* = 0.3934) and risk allele containing genotypes CC (OR: 1.7262 CI: 0.6016–4.9532, *p* = 0.3101) and GC (OR: 1.5517 CI: 0.6165–3.9056, *p =* 0.3508) were statistically not significantly associated with breast cancer risk. In contrast, the risk allele of *OPG* (rs3102735) C (OR: 0.4882 CI: 0.2959–0.8057, *p =* 0.005) and respective genotypes CC (OR: 0.2365 CI: 0.0696–0.8032, *p* = 0.0208) and TC (OR: 0.4138 CI: 0.1991–0.8599, *p* = 0.0181) were statistically significant. Similarly, in case of *RANKL* (rs9533156), the frequency of risk allele C (OR: 2.5887 CI: 1.5924–4.2082, *p =* 0.0001) and respective genotypes CC (OR: 8.1818 CI: 2.7796–24.0836, *p =* 0.0001) and TC (OR: 3.7500 CI: 1.4028–10.0243, *p =* 0.0084) were significantly associated with disease risk (Table 3).

**Table 3:** Allelic and genotypic frequencies of *OPG* and *RANKL* in breast cancer vs healthy controls.

Genotypes/Alleles	BC	HC	BC vs HC	*p-*value
***OPG* (rs2073618)**	**n = 106(%)**	**n = 51(%)**	**OR 95%CI**	
GG	14 (13.21)	10 (19.60)		
GC	63 (59.43)	29 (56.86)	1.5517(0.6165–3.9056)	0.3508
CC	29 (27.35)	12 (23.53)	1.7262(0.6016–4.9532)	0.3101
G	91 (42.9)	49 (48.0)		
C	121 (57)	53 (51.9)	1.2293(0.7651–1.9751)	0.3934
***OPG* (rs3102735)**				
TT	58 (54.72)	16 (31.37)		
TC	42 (39.62)	28 (54.90)	0.4138(0.1991–0.8599)	0.0181
CC	6 (5.66)	7 (13.72)	0.2365(0.0696–0.8032)	0.0208
T	158 (74.5)	60 (58.8)		
C	54 (25.0)	42 (41.0)	0.4882(0.2959–0.8057)	0.005
***RANKL* (rs9533156)**				
TT	8 (7.55)	15 (29.41)		
TC	50 (47.17)	25 (49.02)	3.7500(1.4028–10.0243)	0.0084
CC	48 (45.28)	11 (21.57)	8.1818(2.7796–24.0836)	0.0001
T	66 (31.1)	55 (53.9)		
C	146 (68.8)	47 (46.0)	2.5887(1.5924–4.2082)	0.0001

### *OPG* (rs3102735) and *RANKL* (rs9533156) exhibited risk association with breast cancer to bone metastasis while *OPG* (rs2073618) didn’t show association compared with healthy controls

The genotypic and allelic frequencies of *OPG* and *RANKL* in bone metastasis and healthy controls were investigated (Table 4). The results indicated that *OPG* (rs2073618), risk allele C (OR: 1.0990 CI: 0.6448–1.8731, *p* = 0.7282) and risk allele containing genotypes CC (OR: 1.2963 CI: 0.3961–4.2421, *p =* 0.6679) and GC (OR: 1.3410 CI: 0.4805–3.7422, *p =* 0.5752) didn’t show statistically significant association. Unlikely, *OPG* (rs3102735) indicated that risk allele C (OR: 1.8857 CI: 1.1000–3.2327, *p* = 0.0211) and genotypes CC (OR: 4.5714 CI: 1.3382–15.6164, *p =* 0.0153) and TC (OR: 2.4286 CI: 0.9068–6.5041, *p =* 0.0775) were significantly associated with bone metastasis. Similarly, the *RANKL* (rs9533156) risk allele C (OR: 5.000 CI: 2.7280–9.1642, *p =* 0.0001) and homozygous mutant genotype CC (OR: 11.1818 CI: 3.3299–37.5486, *p =* 0.0001) were significantly associated with high risk of breast to bone metastasis but the heterozygous genotype TC (OR: 1.4400 CI: 0.4234–4.8972, *p =* 0.5593) was not associated with disease risk.

**Table 4:** Frequencies of *OPG* and *RANKL* polymorphisms in bone metastasis vs healthy controls.

Genotypes/Allele	BM	HC	BM vs HC	*P*-value
***OPG* (rs2073618)**	**n = 58(%)**	**n = 51(%)**	**OR (95%CI)**	
GG	9 (15.52)	10 (19.60)		
GC	35 (60.34)	29 (56.86)	1.3410(0.4805–3.7422)	0.5752
CC	14 (24.14)	12 (23.53)	1.2963(0.3961–4.2421)	0.6679
G	53 (45.6)	49 (48.0)		
C	63 (54.1)	53 (51.9)	1.0990(0.6448–1.8731)	0.7282
***OPG* (rs3102735)**				
TT	8 (13.79)	16 (31.37)		
TC	34 (58.62)	28 (54.90)	2.4286(0.9068–6.5041)	0.0775
CC	16 (27.59)	7 (13.72)	4.5714(1.3382–15.6164)	0.0153
T	50 (43.1)	60 (58.8)		
C	66 (56.8)	42 (41.0)	1.8857(1.1000–3.2327)	0.0211
**HWE**	0.331393	0.635496		
***RANKL* (rs9533156)**				
TT	5 (8.62)	15 (29.41)		
TC	12 (20.69)	25 (49.02)	1.4400(0.4234–4.8972)	0.5593
CC	41 (70.69)	11 (21.57)	11.1818(3.3299–37.5486)	0.0001
T	22 (18.9)	55 (53.9)		
C	94 (81.0)	47 (46.0)	5.000(2.7280–9.1642)	0.0001
**HWE**	0.045103	0.995361		

### *OPG* (rs3102735) and *RANKL* (rs9533156) exhibited association with breast to bone metastasis compared with breast cancer patients

The frequencies of *OPG* and *RANKL* in breast cancer and breast to bone metastasis were also checked (Table 5). The results displayed that *OPG* (rs2073618) risk allele C (OR: 1.1186 CI: 0.7094–1.7639, *p =* 0.6295) and risk allele containing genotypes CC (OR: 1.3316 CI: 0.4647–3.8155, *p* = 0.5938) and GC (OR: 1.1571 CI: 0.4548–2.9440, *p =* 0.7593) were not associated with breast to bone metastasis compared with breast cancer patients. Conversely, *OPG* (rs3102735) risk allele C (OR: 0.1704 CI: 0.0716–0.4053, *p* = 0.0001) and genotypes CC (OR: 0.0517 CI: 0.0157–0.1708, *p =* 0.0001) and TC (OR: 0.1704 CI: 0.0716–0.4053, *p =* 0.001) were were strongly associated with breast to bone metastasis. Likewise, *RANKL* (rs9533156) risk allele C (OR: 0.5177 CI: 0.2994–0.8953 *p* = 0.0185) was found significant with disease risk, while the genotypes CC (OR: 0.7317 CI: 0.2220–2.4114, *p* = 0.6077) and TC (OR: 2.6042 CI: 0.7221–9.3922, *p* = 0.1436) were not associated with diseases risk.

**Table 5:** *OPG* and *RANKL* frequencies in breast cancer and breast to bone metastasis.

*OPG* (rs2073618)	BC	BM	BC vs BM	*P-*value
**Genotypes/Alleles**	**n = 106(%)**	**n = 58(%)**	**OR (95%CI)**	
GG	14 (13.21)	9 (15.52)		
GC	63 (59.43)	35 (60.34)	1.1571(0.4548–2.9440)	0.7593
CC	29 (27.35)	14 (24.14)	1.3316(0.4647–3.8155)	0.5938
G	91 (42.9)	53 (45.6)		
C	121 (57)	63 (54.1)	1.1186(0.7094–1.7639)	0.6295
***OPG* (rs3102735)**				
TT	58 (54.72)	8 (13.79)		
TC	42 (39.62)	34 (58.62)	0.1704(0.0716–0.4053)	0.001
CC	6 (5.66)	16 (27.59)	0.0517(0.0157–0.1708)	0.0001
T	158 (74.5)	50 (43.1)		
C	54 (25.0)	66 (56.8)	0.1704(0.0716–0.4053)	0.0001
***RANKL* (rs9533156)**				
TT	8 (7.55)	5 (8.62)		
TC	50 (47.17)	12 (20.69)	2.6042(0.7221–9.3922)	0.1436
CC	48 (45.28)	41 (70.69)	0.7317(0.2220–2.4114)	0.6077
T	66 (31.1)	22 (18.9)		
C	146 (68.8)	94 (81.0)	0.5177(0.2994–0.8953)	0.0185
**HWE**	0.58834	0.045103		

## Discussion

Breast cancer is also one of the frequently diagnosed cancers in woman of Pashtun population of Khyber Pakhtunkhwa, Pakistan. Unfortunately, there is very little work conducted so far, to explore the genetic predisposition of breast cancer and associated metastasis in this region. Here, this study aimed to check the association of *OPG* (rs2073618, rs3102735) and *RANKL* (rs9533156) polymorphism with breast cancer to bone metastasis, as there is no report available for the said population. However, similar studies have been conducted in different ethnicities worldwide to confirm the association of *OPG* and *RANKL* polymorphism with breast cancer to bone metastasis, which displayed contradictory results [24, 27].

Interestingly, our results demonstrated that *OPG* (rs2073618), risk allele C (*p* = 0.3934) and corresponding genotypes GC (*p* = 0.3508 and CC (*p* = 0.3101) didn’t establish significant association with breast cancer risk and also no significant correlation was observed between risk allele C (*p* = 0.7282) and risk allele containing genotypes GC (*p* = 0.5752, CC (*p* = 0.6679) with breast to bone metastasis when compared to healthy controls (Tables 3 and 4), which is in contrast to the previous study conducted in Egyptians and Chinese Han population [10, 14, 28, 29]. Another study conducted among Caucasians (rs2073618), which revealed the minor allele to be protective against breast cancer pathogenesis [30]. However, in the present study individuals carrying homozygous mutant genotype (CC) were at increased risk of breast cancer (OR = 1.7262 95% CI = 0.6016–4.9532) and breast to bone metastasis (OR = 1.3316, 95% CI = 0.4647–3.8155) as compared to the individuals carrying heterozygous genotype. Regarding the *OPG* (rs3102735), the results were found significant between risk allele and risk allele containing genotypes with breast cancer and breast to bone metastasis. Our study result is similar to that of the Caucasian population, where the minor allele C and the corresponding genotypes CC and TC were more frequent in breast cancer patients and showed 1.5-fold increased risk [10]. However, more investigation will be required with large data sets to validate the association of *OPG* polymorphism with breast to bone metastasis.

Concerning the *RANKL* (rs9533156), significant association was observed between the risk allele and corresponding genotypes with breast cancer and breast to bone metastasis. Although, in the current study individuals carrying homozygous mutant genotype (CC) were at increased risk of breast cancer (OR = 8.1818 95% CI = 2.7796–24.0836) as compared to breast to bone metastasis (OR = 0.7317, 95% CI = 0.2220–2.4114). The mutant genotype frequency was found higher among breast cancer than bone metastasis patients. Previous study among Egyptians revealed T the major allele frequently among the breast cancer cases, while the present research found minor allele C repeatedly among breast cancer patients when compared to the healthy controls, which is in contrast to the study done in Caucasian and Egyptian population [10, 14]. A study expressed that minor allele as well as the genotype of the minor allele of the *RANKL* rs9533156 was strongly associated with a higher BMI (>/ = 28) patients with breast cancer group. Whether obese patients carrying the minor allele from one of the two *RANKL* SNPs have an additionally a higher risk of developing breast cancer remains open in this study.

Excluding the contradictory results of genetic polymorphism and risk of breast to bone metastasis in various ethnicities, both *OPG* and *RANKL* have important role in breast cancer to bone metastasis proliferation and progression [31]. However, more investigation will be required to see the exact mechanism of both *OPG* and *RANKL* polymorphism and their association with breast cancer to bone metastasis in various population.

## Conclusion

Our study revealed that *OPG* (rs3102735) and *RANKL* (rs9533156) exhibited significant association with the risk of breast to bone metastasis in Pashtun population, while *OPG* (rs2073618) didn’t show significant association with disease risk. However, more investigation will be required while targeting the full gene sequencing or whole exome sequencing to find the exact situation of genetic predisposition of breast to bone metastasis in Pashtun Population.

## Supporting information

S1 Data (XLSX)

S2 Data (XLSX)

S3 Data (XLSX)

S1 Raw images (PDF)

## References

[1] Bray F, Ferlay J, Soerjomataram I. Global Cancer Statistics 2018: GLOBOCAN Estimates of Incidence and Mortality Worldwide for 36 Cancers in 185 Countries. 2018;394–424. 10.3322/caac.21492 30207593

[2] Menhas R, Umer S. Breast cancer among Pakistani women. Iran J Public Health. 2015;44:586–7. 26056679 PMC4441973

[3] Parkes A, Clifton K, Al-Awadhi A, Oke O, Warneke CL, Litton JK, et al. Characterization of bone only metastasis patients with respect to tumor subtypes. npj Breast Cancer. Springer US; 2018;4:1–7. 10.1038/s41523-018-0054-x PMC5785511 29387785

[4] Xiao W, Zheng S, Liu P, Zou Y, Xie X, Yu P, et al. Risk factors and survival outcomes in patients with breast cancer and lung metastasis: a population-based study. Cancer Med. 2018;7:922–30. doi: 10.1002/cam4.1370 29473333 PMC5852337

[5] Bale R, Putzer D, Schullian P. Local treatment of breast cancer liver metastasis. Cancers (Basel). 2019;11:1–15. doi: 10.3390/cancers11091341 31514362 PMC6770644

[6] Brosnan EM, Anders CK. Understanding patterns of brain metastasis in breast cancer and designing rational therapeutic strategies. Ann Transl Med. 2018;6:163–163. doi: 10.21037/atm.2018.04.35 29911111 PMC5985267

[7] Van Zijl F, Krupitza G, Mikulits W. Initial steps of metastasis: Cell invasion and endothelial transmigration. Mutat Res—Rev Mutat Res. Elsevier B.V.; 2011;728:23–34. doi: 10.1016/j.mrrev.2011.05.002 21605699 PMC4028085

[8] Soni A, Ren Z, Hameed O, Chanda D, Morgan CJ, Siegal GP, et al. Breast cancer subtypes predispose the site of distant metastases. Am J Clin Pathol. 2015; doi: 10.1309/AJCPYO5FSV3UPEXS 25779997

[9] Boyce BF, Xing L. Functions of RANKL/RANK/OPG in bone modeling and remodeling. Arch Biochem Biophys. 2008;473:139–46. doi: 10.1016/j.abb.2008.03.018 18395508 PMC2413418

[10] Ney JT, Juhasz-Boess I, Gruenhage F, Graeber S, Bohle RM, Pfreundschuh M, et al. Genetic polymorphism of the OPG gene associated with breast cancer. BMC Cancer. 2013;13. doi: 10.1186/1471-2407-13-40 23369128 PMC3563620

[11] Weitzmann MN. The Role of Inflammatory Cytokines, the RANKL/OPG Axis, and the Immunoskeletal Interface in Physiological Bone Turnover and Osteoporosis. Scientifica (Cairo). 2013;2013:1–29. 10.1155/2013/125705 PMC3820310 24278766

[12] Sisay M, Mengistu G, Edessa D. The RANK/RANKL/OPG system in tumorigenesis and metastasis of cancer stem cell: Potential targets for anticancer therapy. Onco Targets Ther. 2017;10:3801–10. doi: 10.2147/OTT.S135867 28794644 PMC5538694

[13] Sarink D, Schock H, Johnson T, Overvad K, Holm M, Tjønneland A, et al. Circulating RANKL and RANKL/OPG and breast cancer risk by ER and PR subtype: Results from the EPIC cohort. Cancer Prev Res. 2017;10:525–34. doi: 10.1158/1940-6207.CAPR-17-0125 28701332 PMC5603271

[14] Omar HS, Shaker OG, Nassar YH, Marzouk SA, ElMarzouky MS. The association between RANKL and Osteoprotegerin gene polymorphisms with breast cancer. Mol Cell Biochem. 2015;403:219–29. doi: 10.1007/s11010-015-2352-z 25724681

[15] Santini D, Schiavon G, Vincenzi B, Gaeta L, Pantano F, Russo A, et al. Receptor activator of NF-kB (rank) expression in primary tumors associates with bone metastasis occurrence in breast cancer patients. PLoS One. 2011;6. doi: 10.1371/journal.pone.0019234 21559440 PMC3084800

[16] Fisher JL, Thomas-Mudge RJ, Elliott J, Hards DK, Sims NA, Slavin J, et al. Osteoprotegerin overexpression by breast cancer cells enhances orthotopic and osseous tumor growth and contrasts with that delivered therapeutically. Cancer Res. 2006;66:3620–8. doi: 10.1158/0008-5472.CAN-05-3119 16585187

[17] Hsu YH, Niu T, Terwedow HA, Xu X, Feng Y, Li Z, et al. Variation in genes involved in the RANKL/RANK/OPG bone remodeling pathway are associated with bone mineral density at different skeletal sites in men. Hum Genet. 2006;118:568–77. doi: 10.1007/s00439-005-0062-4 16249885

[18] Roshandel D, Holliday KL, Pye SR, Boonen S, Borghs H, Vanderschueren D, et al. Genetic variation in the RANKL/RANK/OPG signaling pathway is associated with bone turnover and bone mineral density in men. J Bone Miner Res. 2010;25:1830–8. doi: 10.1002/jbmr.78 20205168

[19] Katz J, Gong Y, Salmasinia D, Hou W, Burkley B, Ferreira P, et al. Genetic polymorphisms and other risk factors associated with bisphosphonate induced osteonecrosis of the jaw. Int J Oral Maxillofac Surg. 2011;40:605–11. doi: 10.1016/j.ijom.2011.02.002 21396799

[20] Kim K, Kim JH, Lee J, Jin HM, Lee SH, Fisher DE, et al. Nuclear factor of activated T cells c1 induces osteoclast-associated receptor gene expression during tumor necrosis factor-related activation-induced cytokine-mediated osteoclastogenesis. J Biol Chem. Â© 2005 ASBMB. Currently published by Elsevier Inc; originally published by American Society for Biochemistry and Molecular Biology.; 2005;280:35209–16. doi: 10.1074/jbc.M505815200 16109714

[21] Hu Y, Sun M, Nadiminty N, Lou W, Pinder E, Gao AC. Transcriptional regulation of human RANK ligand gene expression by E2F1. Biochem Biophys Res Commun. Elsevier Inc.; 2008;370:440–4. doi: 10.1016/j.bbrc.2008.03.106 18381203 PMC2950805

[22] Moore LD, Le T, Fan G. DNA methylation and its basic function. Neuropsychopharmacology. Nature Publishing Group; 2013;38:23–38. doi: 10.1038/npp.2012.112 22781841 PMC3521964

[23] Rasmussen KD, Helin K. Role of TET enzymes in DNA methylation, development, and cancer. Genes Dev. 2016;30:733–50. doi: 10.1101/gad.276568.115 27036965 PMC4826392

[24] Geerts D, Chopra C, Connelly L. Osteoprotegerin: Relationship to Breast Cancer Risk and Prognosis. Front Oncol. 2020;10:1–7. 32318347 10.3389/fonc.2020.00462 PMC7154067

[25] Shah M, Danish L, Khan NU, Zaman F, Ismail M, Hussain M, et al. Determination of mutations in iron regulating genes of beta thalassemia major patients of Khyber Pakhtunkhwa, Pakistan. Mol Genet Genomic Med. 2020;8:1–10. doi: 10.1002/mgg3.1310 32588561 PMC7507326

[26] Adnan F, Khan NU, Iqbal A, Ali I, Petruzziello A, Sabatino R, et al. Interleukin-6 polymorphisms in HCC patients chronically infected with HCV. Infect Agent Cancer. Infectious Agents and Cancer; 2020;15:1–7. 10.1186/s13027-020-00285-9 PMC7114800 32266003

[27] Hein A, Bayer CM, Schrauder MG, Häberle L, Heusinger K, Strick R, et al. Polymorphisms in the RANK/RANKL genes and their effect on bone specific prognosis in breast cancer patients. Biomed Res Int. 2014;2014. 10.1155/2014/842452 PMC3963378 24729980

[28] Shaker OG, Senousy MA. Association of SNP-SNP Interactions Between RANKL, OPG, CHI3L1, and VDR Genes With Breast Cancer Risk in Egyptian Women. Clin Breast Cancer [Internet]. Elsevier Inc.; 2019;19:e220–38. Available from: doi: 10.1016/j.clbc.2018.09.004 30309792

[29] Wang J, Lu K, Song Y, Zhao S, Ma W, Xuan Q, et al. RANKL and OPG polymorphisms are associated with aromatase inhibitor-related musculoskeletal adverse events in Chinese Han breast cancer patients. PLoS One [Internet]. 2015;10:1–17. Available from: doi: 10.1371/journal.pone.0133964 26218592 PMC4547828

[30] Assmann G, Juhasz-Boess I, Gruenhage F, Graeber S, Wieczorek S, Pfreundschuh M, et al. Genetic polymorphisms of the OPG gene associated with breast cancer. J Clin Oncol [Internet]. Wolters Kluwer; 2012;30:1523. Available from: doi: 10.1200/jco.2012.30.15_suppl.1523

[31] Infante M, Fabi A, Cognetti F, Gorini S, Caprio M, Fabbri A. RANKL/RANK/OPG system beyond bone remodeling: involvement in breast cancer and clinical perspectives. Available from: doi: 10.1186/s13046-018-1001-2 30621730 PMC6325760

