# Supplementary figures and images for: Risk association of RANKL and OPG gene polymorphism with breast cancer to bone metastasis in Pashtun population of Khyber Pakhtunkhwa, Pakistan
Source: PLoS One. 2022 Nov 8;17(11):e0276813. doi: 10.1371/journal.pone.0276813 (PMC9642875; doi:10.1371/journal.pone.0276813)

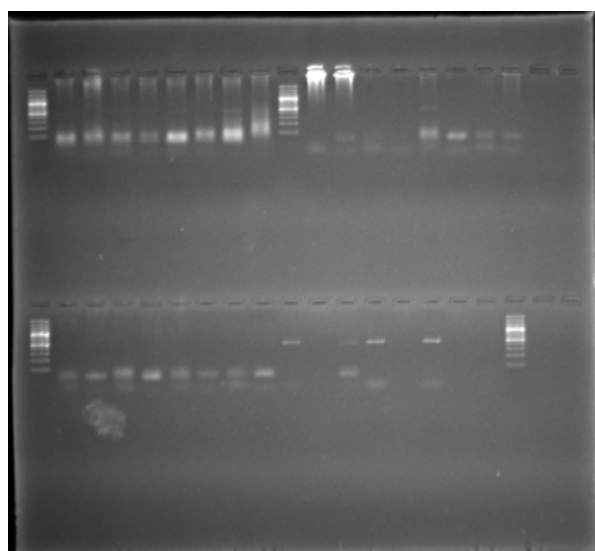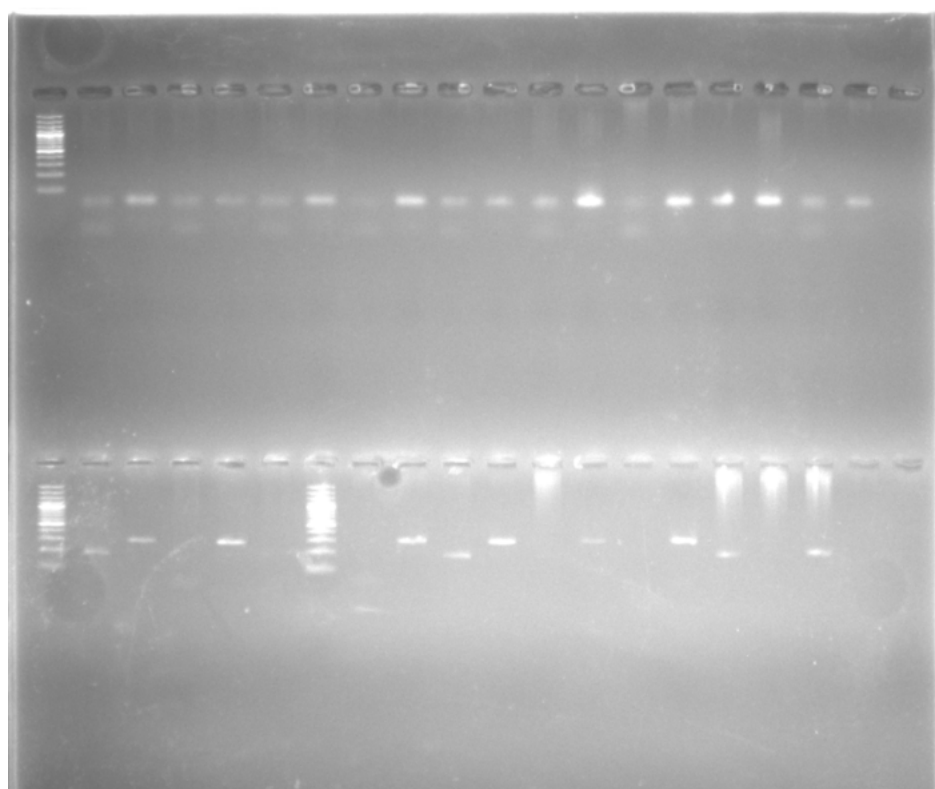

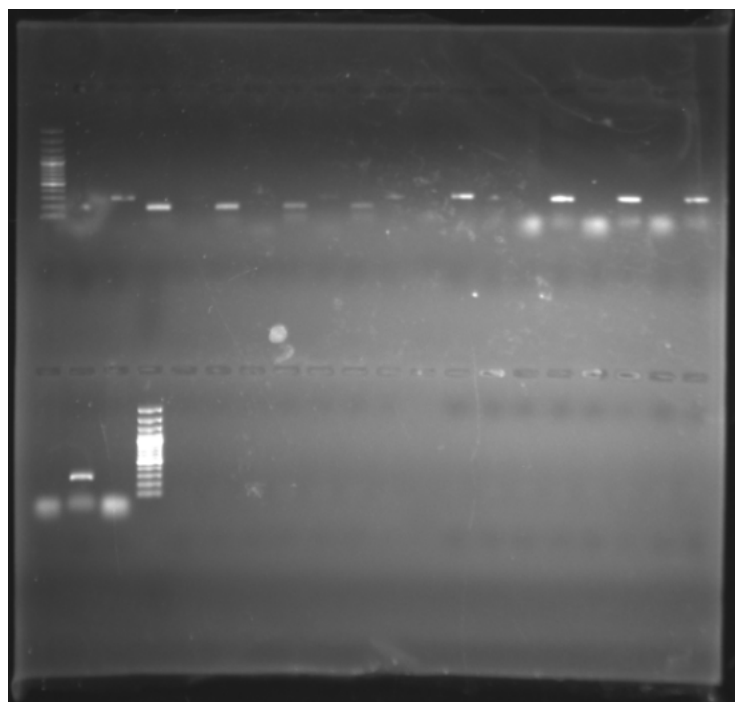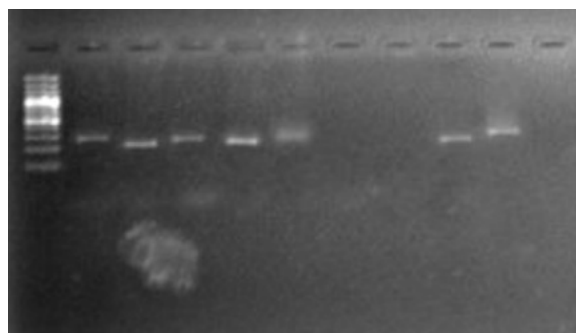

Supplement: S1 Raw images — (PDF) [file pone.0276813.s004.pdf]
